# A saponin-polybromophenol antibiotic (CU1) from *Cassia fistula* Bark Against Multi-Drug Resistant Bacteria Targeting RNA polymerase

**DOI:** 10.1016/j.crphar.2022.100090

**Published:** 2022-02-03

**Authors:** Asit Kumar Chakraborty, Sourajit Saha, Kousik Poria, Tanmoy Samanta, Sudhanshu Gautam, Jayanta Mukhopadhyay

**Affiliations:** a Department of Biotechnology and Biochemistry, Oriental Institute of Science and Technology, Vidyasagar University, Midnapore 721102, India; b Department of Chemistry, Bose Institute, Kolkata 700009, India; c Department of Basic Sciences, IIT-Mandi, Himachal Pradesh 175005, India; d Molecular Biophysical Unit, Indian Institute of Science, Bangalore 560012, India

**Keywords:** Saponin polybromophenol phyto-antibiotic, Cassia fistula bark, RNA polymerase Inhibition, MDR-Bacteria, Anti-TB drug: Antibiotics void, AMR, Antimicrobial resistance, MDR, Multi-drug resistant, Bla, beta-lactamase, BSA, Bovine serum albumin, EDTA, Ethylene di-amine tetra-acetic acid, Rif, Refampicin, FTIR, Fourier transformed Infrared Spectroscopy, NMR, Nuclear Magnetic Resonance Spectroscopy, DTT, Dithiothreitol, EMSA, Electrophoretic Mobility Shift Assay, Mtb, Mycobacterium tuberculosis, DMSO, Dimethyl sulfoxide, HPLC, HighPerformance Liquid Chromatography, PAGE, Poly-Acrylamide Gel Electrophoresis, ppm, parts per million, Rp0, RNA polymerase open complex

## Abstract

**Background:**

Gradual increase of multidrug resistant infections is a threat to the human race as MDR plasmids have acquired.>10 mdr and drug efflux genes to inactivate antibiotics. Plants secret anti-metabolites to retard growth of soil and water bacteria and are ideal source of antibiotics.

**Purpose:**

Purpose of the study is to discover an alternate phyto-drug from medicinal plants of India that selectively kills MDR bacteria.

**Methods:**

MDR bacteria isolated from Ganga river water, milk, chicken meat and human hair for testing phyto-extracts. Eighty medicinal plants were searched and six phyto-extracts were selected having good antibacterial activities as demonstrated by agar-hole assays giving 15 ​mm or greater lysis zone. Phyto-extracts were made in ethanol or methanol (1:5 w/v) for overnight and were concentrated. Preparative TLC and HPLC were performed to purify phytochemical. MASS, NMR, FTIR methods were used for chemical analysis of CU1. In vitro RNA polymerase and DNA polymerase assays were performed for target identification.

**Results:**

CU1 belongs to a saponin bromo-polyphenol compound with a large structure that purified on HPLC C_18_ column at 3min. CU1 is bacteriocidal but three times less active than rifampicin in Agar-hole assay. While in LB medium it shows greater than fifteen times poor inhibitor due to solubility problem. CU1 inhibited transcription from Escherichia coli as well as Mycobacterium tuberculosis RNA Polymerases. Gel shift assays demonstrated that CU1 interferes at the open promoter complex formation step. On the other hand CU1 did not inhibit DNA polymerase.

**Conclusion:**

Phyto-chemicals from Cassia fistula bark are abundant, less toxic, target specific and may be a safer low cost drug against MDR bacterial diseases.

## Highlights

• A new saponin-polybromophenol antibiotic (CU1) was isolated from *Cassia fistula* bark ethanol extract and purified by TLC and HPLC.
• The CU1 inhibits MDR bacteria isolated from human, animal, milk and water resources using agar-hole assay but highly refractile to in vitro assays using LB media due to poor solubility of CU1 in water.
• The chemical structure was partially elucidated by Mass, NMR, FT-IR and UV–Vis spectroscopy. It was suggested that triterpine moiety was linked to two polyphenol derivatives with six bromine atoms.
• The CU1 drug inhibits RNA polymerase of *E. coli* and *M. tuberculosis* as compare to conventional drug rifampicin.
• The CU1 phyto-chemical is less water soluble and less toxic in rat and fish models and will be a low cost drug which is resistant to most mdr bacteria.

## Graphical abstract

**Figure:**
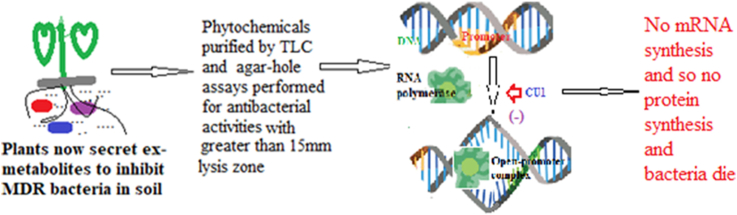

## 1 Introduction

Multidrug-resistant pathogenesis has caused a serious threat to the society where anyone is susceptible to AMR (antimicrobial resistance) disease if immuno-system is weak. AMR disease is a potential threat to life as most common antibiotics (ampicillin, streptomycin, chloramphenicol, ciprofloxacin, cefotaxime, erythromycin, etc) are not able to kill bacteria due to the presence of *mdr* genes (*bla, amp, cat, acc, aad, str, aph, sul, mcr, erm, cfr, dhfr*) in plasmids followed by chromosomal mutations (gyrAB, porB, L4, and rRNA) (McArthur et al., 2013; Chakraborty, 2016a, Chakraborty, 2016b, Chakraborty, 2016c). Few dozens *mdr* genes were isolated and sequenced, and many other mutations with potential higher drug inactivating capabilities were documented, causing human infections by *Salmonella, Pseudomonas, Acinetobacter, Klebsiella, Bacillus,* and *Escherichia* species (Bush et al., 1995; Chakraborty, 2016a, Chakraborty, 2016b). Importantly, small R-plasmids with 1–3 *mdr* genes have combined with F′-conjugative plasmid (62.5 ​kb) giving more space to multiple (5–15) *mdr* genes as well as drug-efflux genes and many (8–15) recombinases and IS-elements. Such bacteria can donate the *mdr* genes to other environmental bacteria by conjugation. Likewise, we found 45% ampicillin resistance bacteria in Ganga River water and Digha sea water whereas reported clinical isolates were >95% ampicillin resistant (Chakraborty, 2017a, Chakraborty, 2017b). In this light drug discovery against these multidrug-resistant bacterial strains from household medicinal plants is quite significant. (Chakraborty., 2015, 2017c; Chakraborty, 2019a, Chakraborty, 2019b).

*Cassia fistula* (Fabaceae family) is a medium sized deciduous tree (10–20m hight and 1–2m girth) cultivated in India for medicinal and decorative purposes (Bhalerao and Kilkar, 2012). The different parts (flower, root, bean, ripe fruit pulp, seed and leaves) are used in curing variety of infections and ailments (Bhalodia et al., 2012). A 3.5KD small protease inhibitor (Fistulin) was purified from leaves of *Cassia fistula* having anti-microbial activities (Rajagopal et al., 2010). Many chemical constituents have been analyzed from *Cassia* bean and tree parts (Sircar et al., 1970; Ginde et al., 1970; Arulpandi et al., 2012; Vaishnav et al., 1993; Bahorun et al., 2005; Bhandari et al., 2013). We first reported the role of *Cassia fistula bark e*xtract in inhibiting superbugs (Chakraborty, 2015). In this study we have showed that RNA polymerase was the target of the purified active chemical. There is only one RNA polymerase in bacteria but four different classes in human (Burgess et al., 1979; Kedinger et al., 1974; Goldberg et al., 1977). The transcription apparatus in eukaryotic organisms is complex (Chakraborty and Hodgson., 1998).

Bacterial RNA polymerase was inhibited by anti-tuberculosis drug rifampicin but in MDR-TB cases it was rendered ineffective. Rif resistant MDR TB carrys the substitution at the beta subunit located near active centre. Having been influenced by herbal-drugs of ancient Indian Hindu civilization described in Sanskrit books Charaka Samhita, Sasruta Samhita and Atharva Veda, we have tested the efficacy of few extracts of medicinal plants including *Cassia fistula* against bacteria. We described here the purification and characterization of the phyto-chemical CU1 from *Cassia fistula* bark that interact with RNA polymerase, inhibiting open-complex formation and thereby RNA synthesis. We have deposited the data to preprint server BioRxiv (2020).

## 2 Material and methods

### 2.1 Collection of bark and chemical extraction

We isolated 8 ​mm thick bark from a medium sized tree (8m). We cut 5 ​mm ​× ​5 ​mm sized pieces of bark with the help of a knife and dried. The extraction was done with ethanol (1:5; w/v) overnight. We recovered ∼11g solid chemicals from 200g bark in 1000 ​ml extraction and evaporation (Chakraborty, 2017d).

### 2.2 Preparative Thin Layer Chromatography

We used 20 ​× ​15cm eight Silica gel plates for purification using 100 ​ml solvent (methanol:water:acetic acid; 50:40:10) in a glass jar with cover lid. It was found that CU1 chemical ran fast near solvent front and get clustered with lines with slow moving due to association of chemicals. From stock dry mass, we dissolved 1g solid in 10 ​ml ethanol, clarified by 10000 ​rpm for 5min and loaded onto silica plate (1 ​cm wide). The ascending chromatography was done for 65min and the plates were dried at room temperature. The gray areas were scratched and suspended in 20 ​ml ethanol and another TLC was run with the dissolved chemical. The dark chemical was scraped again, and ultimately dried at room temperature (60 ​mg CU1 recovered per preparation). Likewise about 95–99% pure CU1 was obtained after repeated TLC (Chakraborty, 2017a).

### 2.3 High performance liquid chromatography

TLC-purified active sample (5 ​mg) was dissolved in 0.5 ​ml methanol, filtered through a membrane filter and 0.1 ​ml sample was loaded onto a HPLC C-18 column equilibrated with methanol. Fractions of 0.5 ​ml were collected and major active peak (retention time-3min) was collected and vacuum dried (Naidoo et al., 2018).

### 2.4 Assay for anti-bacterial activities

We routinely used *E. coli-*KT-1_mdr and *E. coli DH5a* for assays. The E. coli KT-1 bacteria were resistant to ampicillin, ciprofloxacin, sulfamethoxazole, linezolid, vancomycin and moderate resistant to chloramphenicol, cefotaxime and streptomycin, but sensitive to tigecycline and meropenem (Chakraborty., 2017a).

For Kirby-Bauer Agar Hole Diffusion Assays, 100 ​μl overnight culture of MDR-bacteria was spread onto 10 ​cm LB+ 1.5% Agar plate and 6 ​mm holes were done and 40 ​μl ethanolic solution of phyto-chemical was added. In blank we loaded 40 ​μl ethanol which usually did not interfere with the assay, but rarely few strains of MDR bacteria gave minor lysis zone which likely was recovered (re-growth) during extended assay period. We determined >15 ​mm lysis zone was good anti-bacterial activity where 20–30 ​mm lysis zone could be detected for standard 20 ​μl of chloramphenicol (34 ​mg/ml) or streptomycin (50 ​mg/ml) or meropenem (1 ​mg/ml) (Chakraborty., 2015). Different concentration of CU1 or rifampicin added to hole and incubated at 37 ​°C for over noght to get lysis zone. Very low concentration of drugs did not produce lysis zone enough to get a straint line of inhibition.

For in vitro antibacterial assays, we added 0.1 ​ml over night culture of bacteria to 1 ​ml LB media and then added sereially diluted drugs in 20% ethanol and incubated at 37 ​°C for 4 ​h. The OD_600_/ml was taken in a colorimeter and plotted.

#### 2.4.1 Phyto-chemicals assays

TLC-purified chemicals were used for biochemical assays. TLC-purified CU1 chemical was dissolved in water and if ethanol extract was used then ethanol concentration was lowered by adding water during assay. *Assay of Saponins*: 0.5 ​ml plant extract + 2 ​ml methanol + 2 ​ml water. Formation of persistent foam on the surface was taken as an indication for saponin. *Assay of Tannins*: 2 ​ml bromine water + 0.3 ​ml plant extract. Decolouration indicates tannins, *Assay of Flavones*: Alkaline Reagent Test: 2 ​ml of 2% NaOH solution was mixed with 0.4 ​ml plant extract. A concentrated yellow colour was produced, which became colourless with addition of diluted HCl to mixture. *Test for glycosides*: Liebermann's Test: Added 2 ​ml of acetic acid and 2 ​ml of chloroform with plant extract. The mixture was cooled and concentrated H_2_SO_4_ was added. Green colour showed the entity of aglycone and glycosides. *Test for Terpenoids*: 0.5 ​ml chloroform + 0.5 ​ml sulfuric acid concentrated + 0,5 ​ml plant extract + 0.5 ​ml water. Boiled the mixture. A gray colour indicates triterpenes. *Test for steroids*: 0.4 ​ml chloroform + 1 ​ml plant extract ​+ ​few drop concentrated sulfuric acid. Red colour indicates steroids. *Test for alkaloids*: Alcohol extracts were diluted with dilute HCl (1:5v/v) and treated with few drops of Mayer's reagent. A reddish brown or orange precipitate indicates the presence of alkaloids (Harborne et al., 1999; Sambrook et al., 1989).

### 2.5 Fourier transformed infra red spectroscopy

HPLC-purified dry active chemical 5 ​mg was mixed with 200 ​mg IR-grade KBr and the tablet was prepared at 13 ​mm Die SET (Kimaya Engineers) at 10 ​kg/cm^2^. Spectra were taken with a Perkin Elmer Spectrum 100 FT-IR Spectrometer (Serial no. 80944) for 10 ​min (Griffiths and de Hasseth, 2007).

### 2.6 NMR spectrometry

Proton-NMR spectrometry was performed in CDCl_3_, D_2_O and CD_3_OD (4 ​mg/ml CU1) for 10min. Carbon-NMR was performed in D_2_O only (20 ​mg/ml CU1) in 500 ​MHz JNM-ECX500 Spectrometer (Chakraborty, 2019a, Chakraborty, 2019b).

#### 2.6.1 Assay of Escherichia coli RNA Polymerase using ^3^H-UTP

The assay used to measure RNAP activity was performed as described by Lowe et al. (1979) with minor modification (Lowe et al., 1977; Mukhopadhyay et al., 2003). The reaction (Rx 40 ​μl) was performed with transcription buffer (40 ​mM Tris-HCl pH 7.9, 200 ​mM NaCl, 10 ​mM MgCl_2_, 0.1 ​mM EDTA, 14 ​mM β-ME, 200 ​nM each ATP, GTP and CTP. 50 ​μM UTP, 2 ​μCi ^3^H-UTP (BRIT, Hyderabad, India), 1.5 μg calf thymus DNA and 1U RNA polymerase at 37 ​°C for 20 ​min. The reaction mixture was spotted onto DEAE-paper pre-socked with 5 ​mM EDTA. The filter paper was then washed with 5% di-sodium hydrogen phosphate, thrice with water and finally with 95% ethanol and dried. The filters were placed into scintillation vials containing 10 ​ml toluene-based scintillation fluid and counts were recorded on a Tri-CARB 2900 ​TR scintillation counter. Data was normalized and plotted. Rifampicin (10 ​mg/ml in basic ethanol) was used as standard drug and 11 phyto-chemicals (4 times TLC purified; ∼10 ​mg/ml in DMSO) were used.

### 2.7 Mycobacterium RNA polymerase and recombinant σ^A^ purification and the bacterial strains used

*E. coli* strains BL21 (DE3) and C43 (DE3) have been used for the purification of *Mtb* RNA polymerase (RNAP) σ^A^holo and *Mtb.*σ^A^ respectively. For the purification of these proteins, respective *E. coli* cells have been transformed with their respective plasmids and were plated. The colonies obtained were then cultured in 2xYT medium (16g Tryptone, 10g Yeast Extract, 5g NaCl, pH 7.0) in presence of suitable antibiotics. The cultured cells were then pelleted down at 5000 ​rpm in a Sorvall cold centrifuge and lysed to purify the over expressed proteins of interest using specific purification protocols. *In vivo* assembled *Mtb* RNAP holo enzyme was purified as in (Banerjee et al., 2014).

### 2.8 α^32p^- UTP based in vitro transcription assay

Radioactivity based in vitro transcription assay was done as previously described (Banerjee et al., 2014). To perform this assay 300 ​nM Mtb*.*RNAP *σ*^*A*^holo was incubated with 1200 ​nM *σ*^*A*^ in 2 μl transcription buffer (45 ​mM Tris-HCl pH 8.0, 70 ​mM KCl, 1 ​mM DTT, 10% Glycerol, 5 ​mM MgCl_2_ and 1.5 ​mM MnCl_2_) at 25° Celsius for 15 ​min. 200 ​nM DNA template (containing sinP3 promoter) was added to the reactions along with 0, 10, 20, 40, 80, 160 ​μg/ml of the phyto-chemical CU1, and incubated at 37° Celsius for 15 ​min to form open promoter complex (RPo). Heparin (0.25 ​mg/ml) was added to the reactions to inhibit non-specific RNAP-DNA complex. RNA synthesis was initiated by addition of NTP mix (final concentration 250 ​μM of ATP, GTP and CTP and 10 ​μM UTP containing 5 ​μCi α32p- UTP) and incubated for elongation at 37° Celsius for 30 ​min. The reactions were terminated by addition of 2 μl FLB dye (80% formamide, 10 ​mM EDTA, 0.01% Bromophenol Blue, 0.01% Xylene Cyanol). Products were heated at 95° Celsius for 5 ​min, chilled on ice, and resolved by 12% Urea-PAGE, then scanned by storage phosphor scanner (Typhoon trio+, GE Healthcare) and quantified using Image Quant TL software.

### 2.9 Fluorescence based in vitro transcription assay

Fluorescence based in vitro transcription assay was described (Datta et al., 2016). 400 ​nM Mtb*.* RNAP*σ*^*A*^holo was incubated in 2 μl transcription buffer (50 ​mM Tris-HCl (pH 8), 100 ​mM KCl, 10 ​mM MgCl_2_, 1 ​mM DTT, 50 ​nM BSA and 5% glycerol) at 25° Celsius for 15 ​min to reactivate. 5 ​ng/μl of pUC19-sinP3 plasmid DNA along with 0, 10, 20, 40, 80 ​μg/ml of the phyto-chemical CU1, was further added and incubated at 37° Celsius for 20 ​min to form open promoter complex (RPo). RNA synthesis was initiated by the addition of NTP mix (final concentration 250 ​μM of ATP, GTP, CTP and UTP) and incubated for elongation at 37°Celsius for 30 ​min. The reaction was stopped by adding 5U of RNase-free DNase I (Thermo Scientific), followed by incubation for 1 ​h at 37° Celsius. RiboGreen dye (Invitrogen, Carlsbad, CA) diluted to 200 folds in TE buffer (10 ​mM Tris-HCl pH 8.5, 1 ​mM EDTA) was prepared. After DNase I digestion, the samples were incubated for 5 ​min at 25° Celsius with the prepared RiboGreen dye solution in TE buffer, so that the reaction mix get 10-fold diluted. Fluorescence intensities of the samples were monitored using a spectrofluorometer (Photon Technology International, HORIBA Scientific, Edison, NJ) at excitation and emission wavelengths of 500 and 525 ​nm, respectively (Mukhopadhyay et al., 2003).

### 2.10 Electrophoretic Mobility Shift Assay

Electrophoretic Mobility Shift Assay (EMSA) was previously described (Rudra et al., 2015). Cy5 labelled primer for the *sinP3* DNA fragment was used for this assay. The promoter DNA fragment was amplified using PCR and the Cy5 labelled amplified DNA was precipitated with an equal volume of isopropanol and 0.1 volume of 3M Na-acetate (pH 5.3). For the assay, 1.2 μM Mtb*.* RNAP was incubated at 25° Celsius in 2 μl transcription buffer (45 ​mM Tris-HCl (pH 8.0), 70 ​mM KCl, 1 ​mM DTT, 10% Glycerol, 5 ​mM MgCl_2_ and 1.5 ​mM MnCl_2_) for 15 ​min to reactivate. 400 ​nM Cy5 labelled *sinP3* promoter containing DNA fragment and heparin at 0.25 ​mg/ml was then added to reactions along with 0, 5, 10, 20, 40, 80 and 160 ​μg/ml of the phyto-chemical CU1 and incubated at 37° Celsius for 30 ​min to form open promoter complex (RPo). The reactions were then resolved in 5% native PAGE in 1xTBE buffer (89 ​mM Tris base, 89 ​mM Boric Acid, 2 ​mM EDTA) for 90 ​min at 4° Celsius and then scanned and examined by Typhoon trio+ (GE Healthcare) scanner using Cy5 channel.

### 2.11 In vitro replication assay

In vitro replication assay was previously described (Rudra et al., 2015). Single-stranded DNA template pUC19-sinP3 and its complementary Cy5 labelled primer were annealed in annealing buffer (50 ​mM Tris.HCl pH 7.5 and 100 ​mM NaCl) by heating to 95° Celsius followed by gradually cooling down to 25° Celsius. The sequence of the Cy5-sinP3 forward primer is: 5’-/Cy5/CAG CCA GAA GTC ATA CCG-3’. 50 ​nM annealed DNA template was incubated with 0, 10, 20, 40, 80 and 160 ​μg/ml of the phyto-chemical CU1 in respective reactions, at 37° Celsius for 5 ​min 0.2 units of Klenow fragment (NEB) and 0.25 ​mM dNTP mix were added to each reaction and were incubated at 37° Celsius for 1 ​min. The reactions were terminated by addition of 2 μl FLB dye (80% formamide, 10 ​mM EDTA) and heated at 95° Celsius for 2 ​min then chilled on ice. Products were resolved by 12% Urea-PAGE and the gel was scanned in Typhoon trio+ (GE Healthcare) scanner using Cy5 channel.

## 3 Results

### 3.1 Purification of CU1 phyto-chemical

**Fig. 1:**
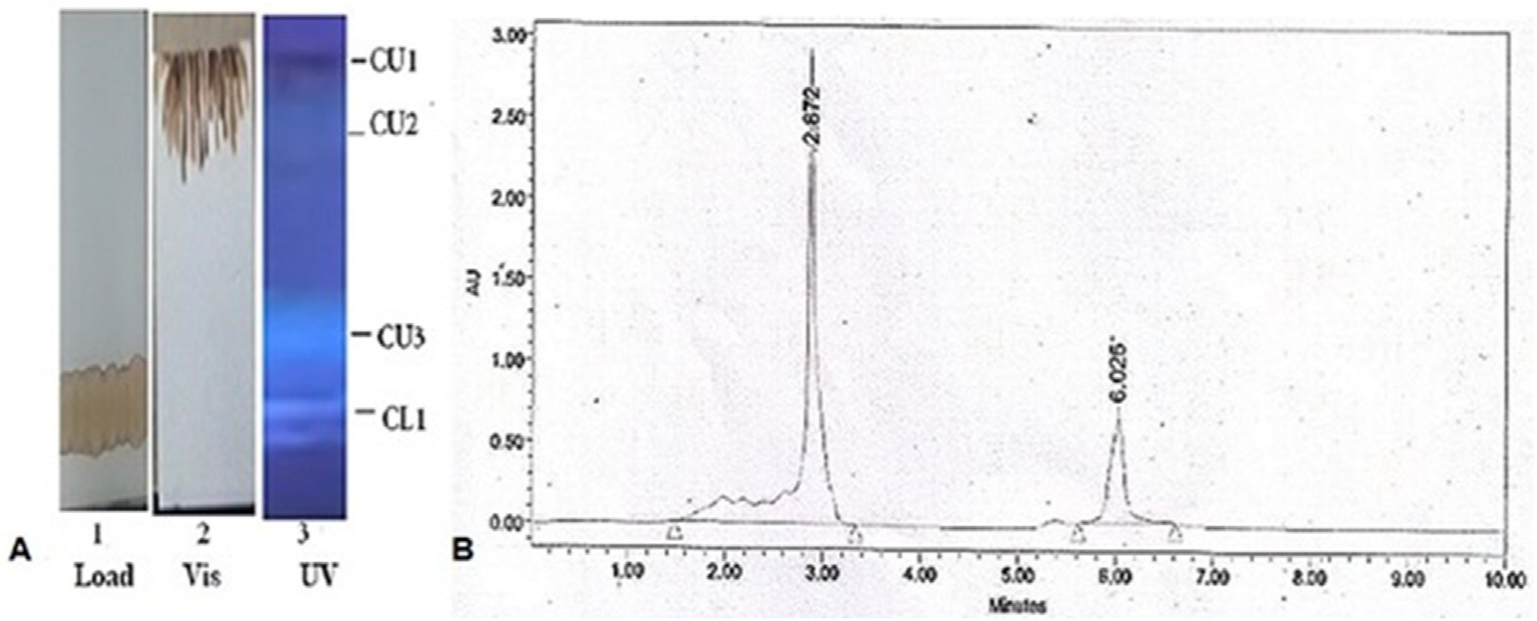
Purification of the CU1 from organic extract of Cassia fistula bark. (A) Preparative Thin Layer Chromatography showing CU1 as gray fast moving major band. 1/6 of the TLC was shown here. CU3 also has anti-bacterial activity and (B) HPLC C_18_ column chromatography showing minor contaminant in TLC preparation and very fast elution time of CU1.

We purified the major phyto-chemicals CU1 from 10 to 12 ​ml soluble ethanol extract using preparative TLC (20 ​× ​15cm 8 plates and four solvent chambers) and 12 times such preparations were done. Further TLC purifications were repeated to obtain pure drug. Usually, we recovered ∼300 ​mg CU1 per TLC purification scheme (figure-1A). We performed such purification scheme 3 times (in five years span) to perform all experiments (∼1.2g CU1). CU1 chemical purified on HPLC C_18_ column at 3min and a minor contaminant at 6min (figure-1B). Very low retention time of CU1 demonstrated a big molecule with hydrophobic nature like triterpenes.

### 3.2 Anti-bacterial activities of CU1 and other phyto-chemicals from *Cassia fistula* bark as compared to rifampicin

Anti-bacterial activity was previously reported for ethanol crude extract of *Cassia fistula* bark (Chakraborty., 2015, 2017a). Anti-bacterial activities of TLC-purified phyto-chemicals CU1 and CU3 were comparable but CU2 was less active. Concentrated extract showed antibacterial activity with 15–20 ​mm lysis zone during Kirby-Bauer assay as compared to 20 ​μl of 34 ​mg/ml chloramphenicol or 20 ​μl of 1 ​mg/ml meropenem (20 ​mm) using *Escherichia coli* KT-1_mdr (accession no. KY769881) and E. coli DH5a which did not carry any mdr gene in its genome and very sensitive to drug (figure-2A). We showed that in agar-hole assay, the CU1 was 2–3 times poor inhibitor (Fig. 2A, panel b) than rifampicin. While in LB media the CU1 appeared less effective drug killing E. coli KT-1 as well as E.coli DH5a due to insolubility of CU1 in water (Fig. 2B). Hence, the MIC determination was not reasonable which appeared much higher than rifampicin (10 ​μg/ml vs. 150 ​μg/ml)! We hope specific addition of –COOH or –NO2 group to triterpine moity may increase the solubility of drug in water and will lower the MIC. MDR E. coli KT-1 showed high resistance due to its huge amplification of acrAB drug efflux gene as previously showed by PCR assay and the bacteria also contained huge large MDR plasmids (Chakraborty, 2017a).

**Fig. 2A:**
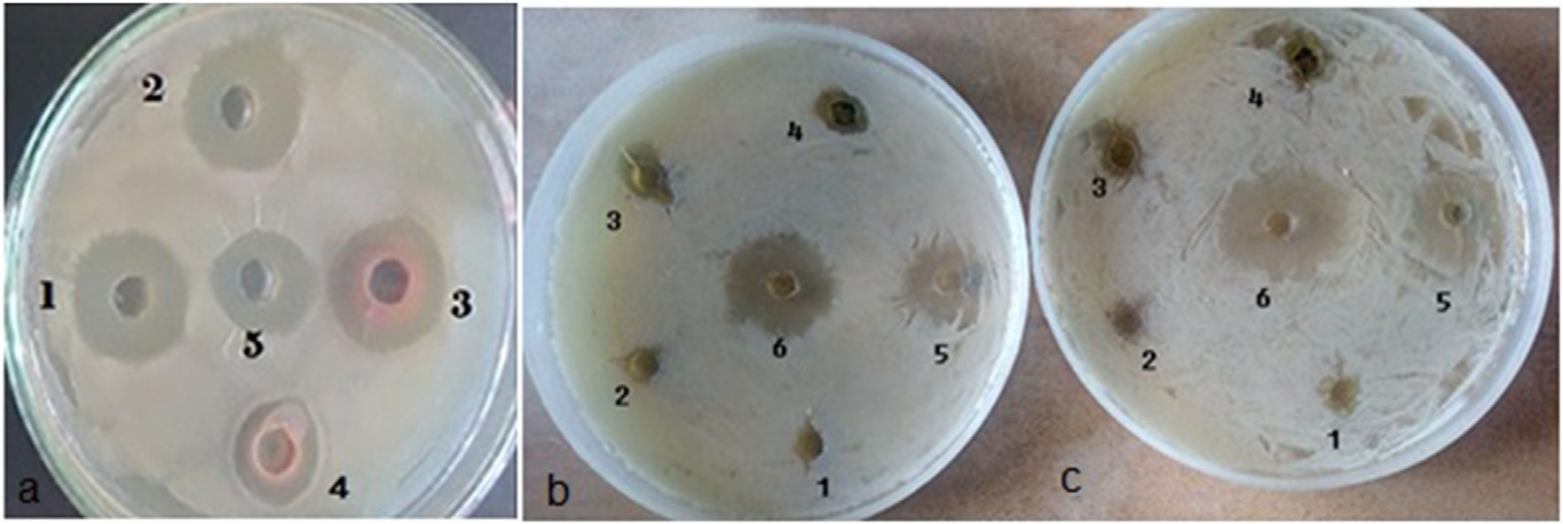
Kirby Bauer Agar Hole Diffusion Assay: (a) TLC purified CU1, Holes: 1. NU3; 2. VU3; 3. CU1; 4. CU1-HPLC purified; 5. Meropenem 20 ​μl (1 ​mg/ml) (b) different concentration of HPLC purified CU1 at 0, 10, 20, 50, 100, 200 ​μg (Holes 1–6) (c) different concentration of Rifampicin at 0, 0.5, 1, 5, 10, 20 ​μg (Holes 1–6).

**Fig. 2B:**
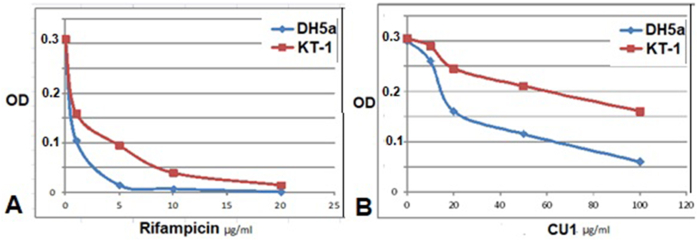
In vitro assay E. coli DH5a and MDR E. coli KT-1 in presence of phyto-chemical CU1 (B) as compared to rifampicin (A).

We searched local area of Midnapore (22.658 Lattitude x 87.593 Longitude) and selected *Cassia fistula* bark (phyto-chemical named CU), *Suregada multiflora* (local name *Narenga*; phyto-chemical named NU) and *Jatropha gossypiifolia* (local name *Varenda*; phytochemical named VU) as valuable sources for new drugs to cure superbug infection where no antibiotics work. (Chakraborty., 2017a). Due to the multi-component nature of chemical structures, such heterogeneous phyto-antibiotics hard to become drug-resistant due to *mdr* genes as well as activation drug-efflux genes in E. coli KT-1.

### 3.3 Characterization of CU1 phyto-chemical and structure prediction

**Fig. 3:**
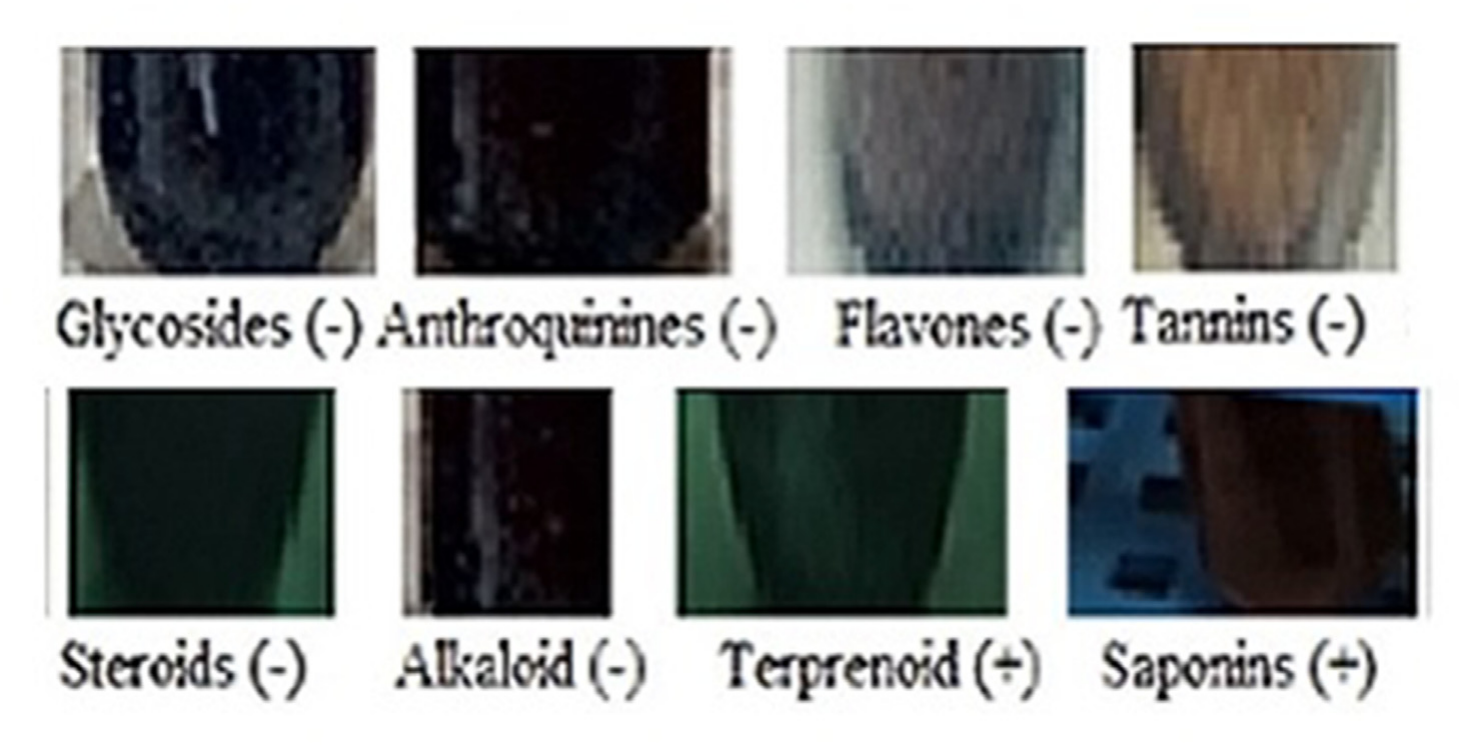
Biochemical assays of TLC purified CU1 phyto-chemical showing ​+ ​ve for terpenetene and saponin.

**Fig. 4:**
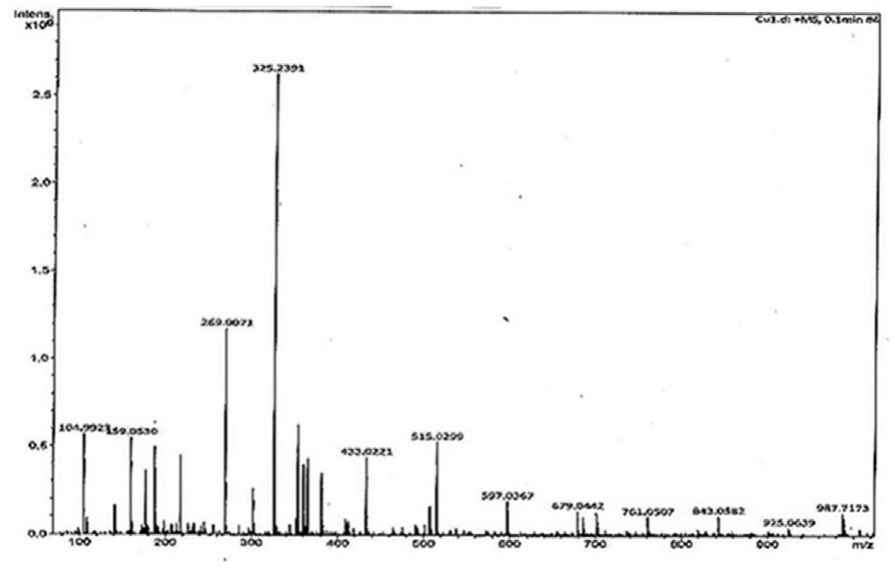
Mass spectra of CU1 phyto-chemical showing 987 ​daltons ​MW. In this method, 100-1000MW mass limit but DBr band was detected at other method showing 79.5 ​Da major fragment.

**Fig. 5:**
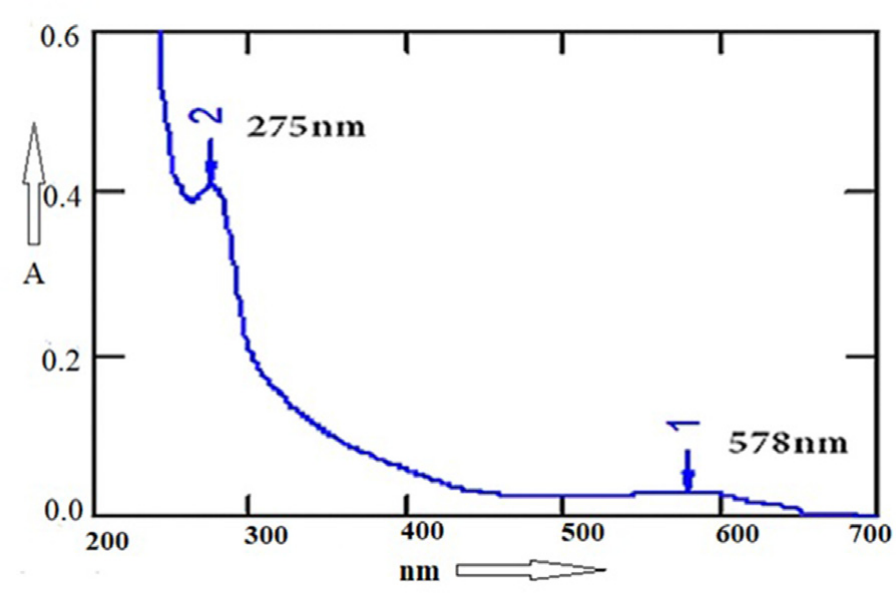
UV–Vis spectroscopy of TLC-purified CU1 showing peaks at 578 ​nm and 275 ​nm.

Chemical analysis also showed it had saponin character as well as triterpenes (figure-3). CHN elementary analysis gave 35.9% carbon and 5.5% hydrogen but no nitrogen indicating a saponin or glycoside. We confused for elementary data as 58.6% oxygen was impossible and we suspected a halogen derivative with 6 bromine atoms giving ∼33.6% Br and ∼25.2% Oxygen could be possible which confirmed by MASS spectrometry. The mass of CU1 was predicted 987.7173 ​Da and from Mass spectra data (figure-4) we concluded that DBr 82 ​MW deviations five derivatives (925.0639, 843.0582, 761.0507, 679.0442, 597.0367, 515.0299, 433.0221 ​Da) suggesting bromine atoms attached to carbon or –OH of polyphenol. CU1 Mass fragment of 325 may be triterpene plus a mono bromo-polyphenol and 987 ​Da mass was calculated as possible dimeric bromo-polyphenol derivative and a 79.5 ​Da major band may reflect Br ​+ ​ion but not shown here (figure-4). VU-Vis spectra gave 275 ​nm peak with minor hinge at 578 ​nm indicating a fused ring with polyphenolic substituent (figure-5). This interpretation was considered as phenolic compound and bromo-benzene both have primary peak at 210 ​nm and secondary peaks at 270 ​nm and 261 ​nm respectively. So high peak at 210 ​nm with secondary peak 275 ​nm was justified for polyphenolic compound with multiple bromine whereas, for turpentine moiety further peak was shifted below 200 ​nm was observed.

FT-IR suggested broad band at 3650-2980 ​cm^−1^ for –OH (stretching, ∼3500 ​cm^−1^) and C–H (stretching, ∼3000 ​cm^−1^) where as two strong peaks at 1552 ​cm^−1^ for aromatic C=C (stretching) and 1408 ​cm^−1^ for phenol (-OH bending). Minor peaks at 2360 ​cm^−1^ with 2340 ​cm^−1^ shoulder represented terpenetine double bond. Similarly, medium peaks at 1246 ​cm^−1^, 1159cm-1, 1105 ​cm^−1^, ∼837 ​cm^−1^ and 651 ​cm^−1^ were detected (figure-6A). The absorptions peaks at 1246cm^-^1, 1159 ​cm^−1^ and 1105 ​cm^−1^ may be for secondary alcohol (-OH bending), at 837 ​cm^−1^ likely aromatic hydrogen and at 651 ​cm^−1^ with 619 ​cm^−1^ shoulder likely represented for different C–Br. However, we did not prepare any derivative yet to confirm structure.

**Fig. 6A:**
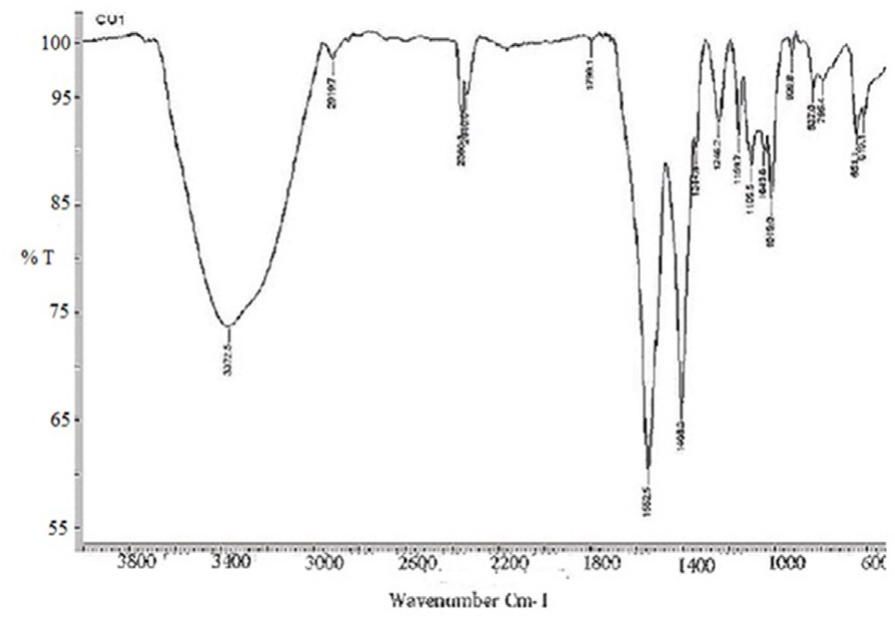
FT-IR spectra of CU1 phyto-chemical. A broad peak at 3000-3500cm-1 for –OH and strong peaks at 1552cm-1 and 1408 ​cm-1are important for phenolics.

Proton-NMR confirmed polymeric phenol at δ 4.86–4.91 ​ppm and tetratet at δ3.57–3.61 ​ppm with phenolic bromo-substituents (figure-6B). Benzoid group confirmed at δ 8.41–8.53 ​ppm where as sharp peaks at δ 3.3 ​ppm and δ 1.88 ​ppm confirmed phenolic bromo substituents (figure-6B). A strong band at δ 8.41–8.53 ​ppm was evident in D_2_O as well as CD_3_OD but in CDCl_3_ H^1^NMR a strong peak at δ7.262 ​ppm and medium peak at δ 1.588 ​ppm were evident (data not shown). However, peak at δ 1.88 ​ppm may link a fused C ​= O in the triterpene and for –CH_2_ at δ 0.11 ​ppm (figure-6B). The δ3.29–3.33 ​ppm bands in CD_3_OD was sharp at δ3.41 ​ppm in D_2_O and δ2.75 ​ppm in CDCl_3_ (data not shown). This difference may reflect differential solubilisation and polar effects as triterpene moity is poor soluble water that polyphenol moity.

**Fig. 6B:**
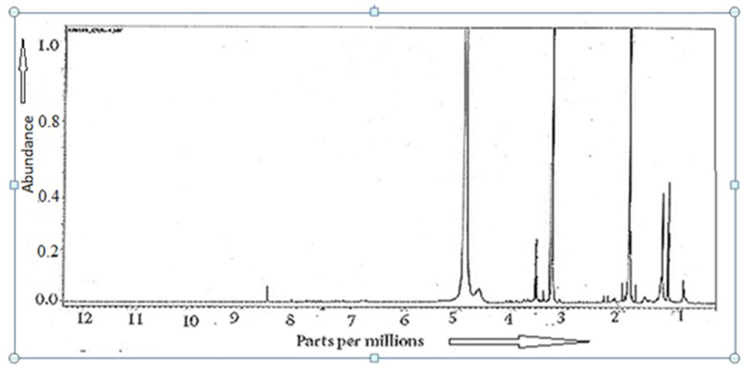
Proton-NMR spectra of CU1 phytochemical from Cassia fistula bark. Peaks at δ ​= ​8.4 ​ppm for polybenzoid and at δ ​= ​4.9 ​ppm for polyphenolic –OH with δ ​= ​3.55 ​ppm for C–Br whereas at δ ​= ​1.88 for C=O absorption in triterpene.

Carbon-NMR identified a strong peak at δ ​= ​23.7 ​ppm for many C–Br and at δ ​= ​165 ​ppm for a polybenzoid compound whereas weak peaks for –CH2- and ​= ​C- carbons of triterpene were detected at δ ​= ​54 ​ppm and 65 ​ppm (figure-6C). More analyses were necessary for complete chemical structure prediction (in process).

**Fig. 6C:**
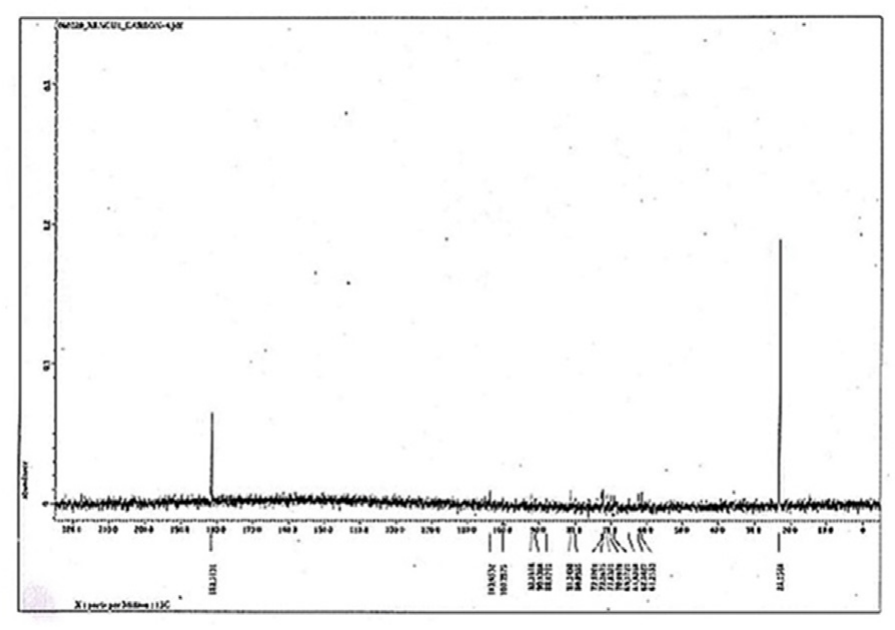
Carbon-NMR spectra of CU1 phyto-chemical. Strong peaks at δ ​= ​23.7 ​ppm for many C–Br and δ ​= ​165 ​ppm for polybenzoid compound are important. Small peaks for –CH2- and ​= ​C- carbons of triterpene were detected at δ ​= ​54–65 ​ppm.

#### 3.3.1 Transcription assays of 11 phyto-chemicals using E. coli RNA polymerase

A multiple-round non-specific transcription assay was performed using calf-thymus DNA as template, 2μci ^3^H-UTP as tracer and *E. coli* RNA polymerase. We found RNA polymerase activity was completely inhibited (complete-1325 cpm, +Rif-63 cpm, +CU1-75 cpm, -Template DNA-52 cpm) only with CU1 and NU2 phyto-chemicals but not with CU3, NU3, VU2, VU3, DU2, LU2, LU3 etc. The inhibitory activity of CU1 was comparable to drug rifampicin and characterized further with priority (figure-7A).

**Fig. 7A:**
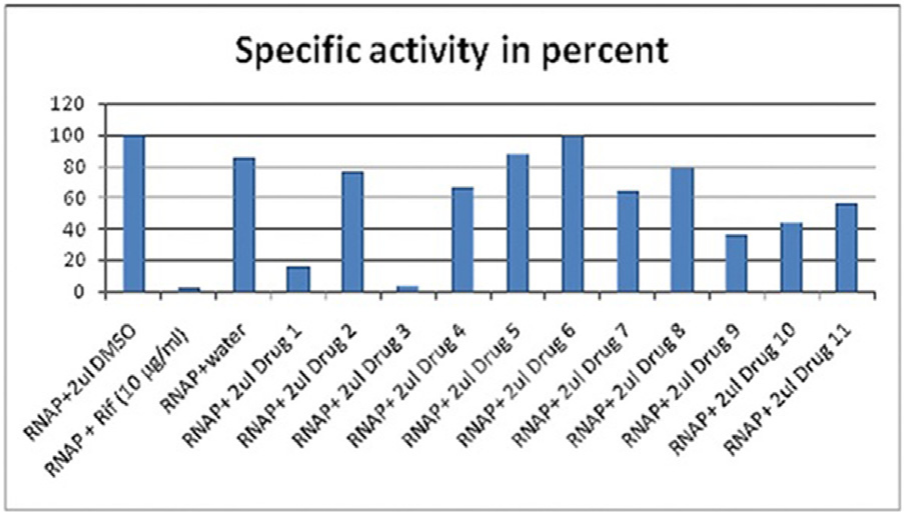
RNA polymerase assays of eleven bioactive phyto-chemicals isolated in our laboratory and discovery of target for CU1 (drug 3) from *Cassia fistula* bark and NU2 (drug 1) from *Suregada multiflora* root. Phytochemical concentrations are ∼10 ​mg/ml and standard is rifampicin (Rif).

#### 3.3.2 Effect of CU1 on RNA polymerases of M. tuberculosis and E. coli

To test the effect of CU1 on transcription by RNAP of *Mycobacterium tuberculosis* a fluorescence based in vitro transcription assay was also performed. Here, the RNA product formed after transcriptional elongation was bound to RiboGreen dye (Invitrogen, Carlsbad, CA), an ultrasensitive fluorescent nucleic acid stain used for quantitating RNA in solution. The fluorescence intensity in each reaction was then measured using a spectrofluorometer (Photon Technology International, HORIBA Scientific, Edison, NJ) at excitation and emission wavelengths of 500 and 525 ​nm, respectively. The results show that CU1 represses the transcriptional activity of *Mtb* RNAP*σ*^*A*^holo as well as *E. coli* RNA polymerase where refampicin is 2–3 time more active than CU1 (figure-7B). Careful estimation was found E. coli RNA polymerase IC50 much less for Rifampicin and much lower for Mtb RNA polymerase (data not shown).

**Fig. 7B:**
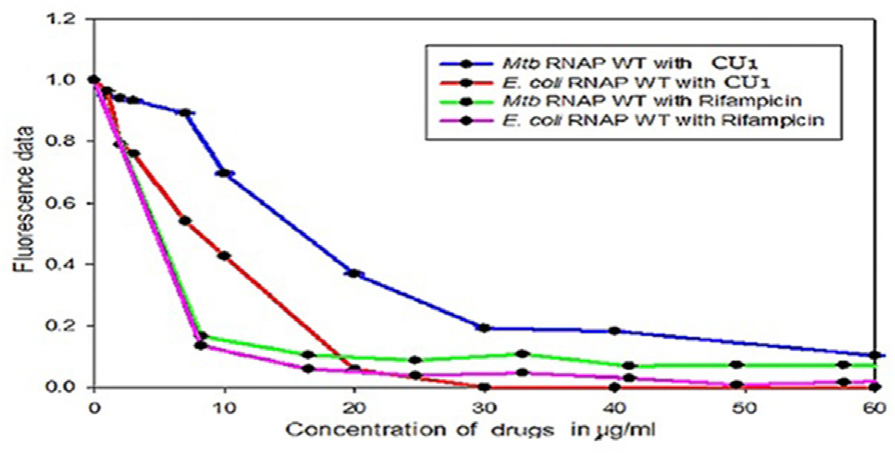
Kinetics of CU1 inhibition of *E. coli* and *M. tuberculosis* RNA polymerases as compared to rifampicin drug.

To further test the transcriptional repression activity of CU1, radioactivity based in vitro transcription assay was performed using α^32p^-UTP. The *sinP3* promoter containing DNA used here generated a run-off transcript of length 70 nucleotides. The RNA products formed, incorporated the radio-labelled NTP present in the NTP mix (α^32p^-UTP) which helped to visualize the products after resolving in a 12% urea-PAGE. The results showed a repression of transcription with an increasing concentration of CU1 (figure-8A).

**Fig. 8A:**
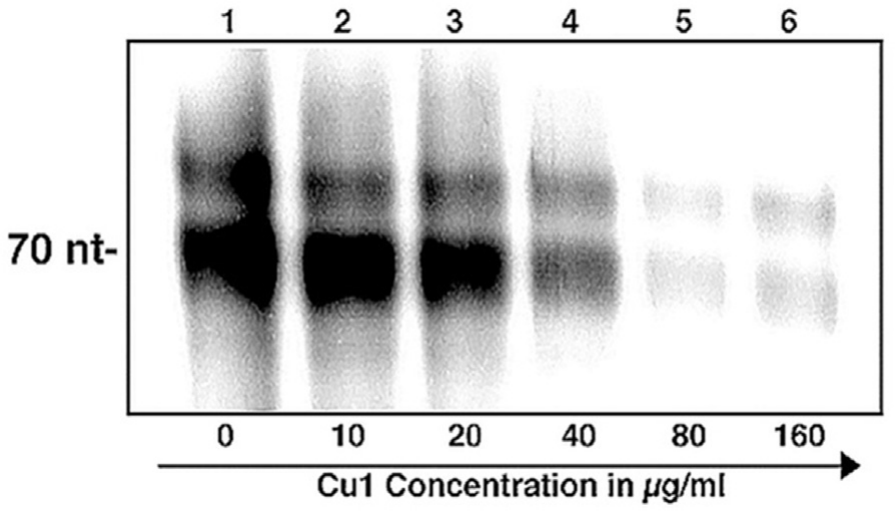
Radioactivity based in vitro transcription assay: 300 ​nM Mtb*.*RNAP *σ*^*A*^holo was incubated with 1200 ​nM *σ*^*A*^, 200 ​nM *sinP3* promoter DNA, along with increasing concentrations of CU1 to form open promoter complex (RP_o_). Radiolabelled-NTP was added to initiate transcription. The estimated IC_50_ value of CU1 was found to be ∼34 ​μg/ml. Concentrations of CU1 used in the reactions (in μg/ml) are shown. Run-off transcript size is 70 ​nt.

The effectiveness of CU1 as a transcriptional inhibitor led to the investigation of its effect on bacterial DNA polymerase activity. For this study pUC19-sinP3 single-stranded DNA template was annealed with Cy5 labelled complementary primer. Then, the primer was allowed to be extended by Klenow fragment (E. coli DNA polymerase; NEB) using the annealed single-stranded DNA as template, in presence of increasing concentrations of CU1. The products were then resolved in a 12% urea-PAGE for visualization. The results show no significant change in DNA replication products formed in the presence of increasing concentrations of CU1 (Figure-8B). This shows that CU1 does not affect bacterial DNA polymerase activity. This also suggested that CU1 did not bind to DNA alone.

**Fig. 8B:**
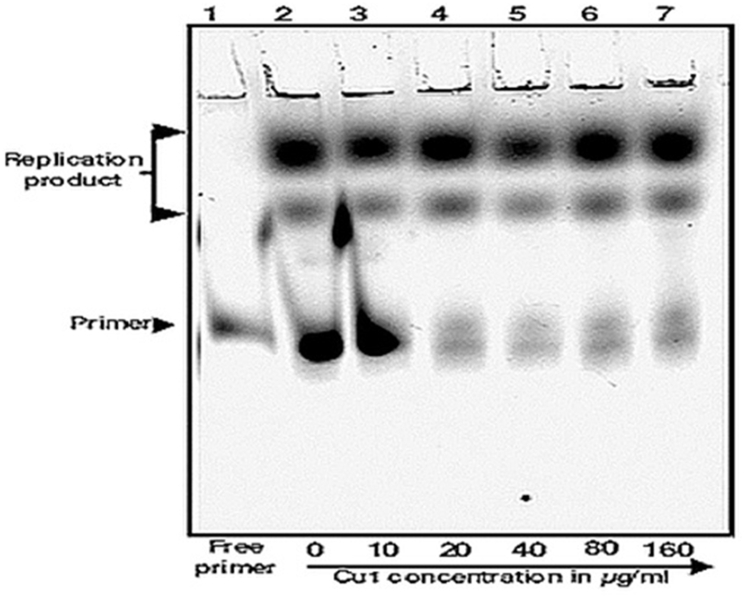
In vitro replication assay with the increasing concentration of CU1. Single-stranded DNA template pUC19-sinP3 and its complementary Cy5 labelled primer were annealed and then incubated with increasing concentration of the repressor CU1(in μg/ml). DNA polymerase, Klenow fragment (NEB) and 0.25 ​mM dNTP mixture were then added to the reaction mixtures to initiate DNA polymerization. Lane 1: Free primer, Lanes 2–7: Replication product formation by Klenow fragment (NEB) in the presence of an increasing concentration of CU1(in μg/ml).

### 3.4 Determination of IC50 and open-complex formation

The estimated IC_50_ value of CU1 was found to be ∼34 ​μg/ml using radioactive assay but much less 23 ​μg/ml in fluorescence assay. The reactions containing 80 and 160 ​μg/ml CU1 the production of radio-labelled RNA was almost completely quenched (figure-9A). The results obtained from these experiments confirmed that CU1 was a strong transcriptional repressor with *Mtb* RNAP. This is very interesting as MDR-TB is very much active in the Indian sub-continents.

**Fig. 9A:**
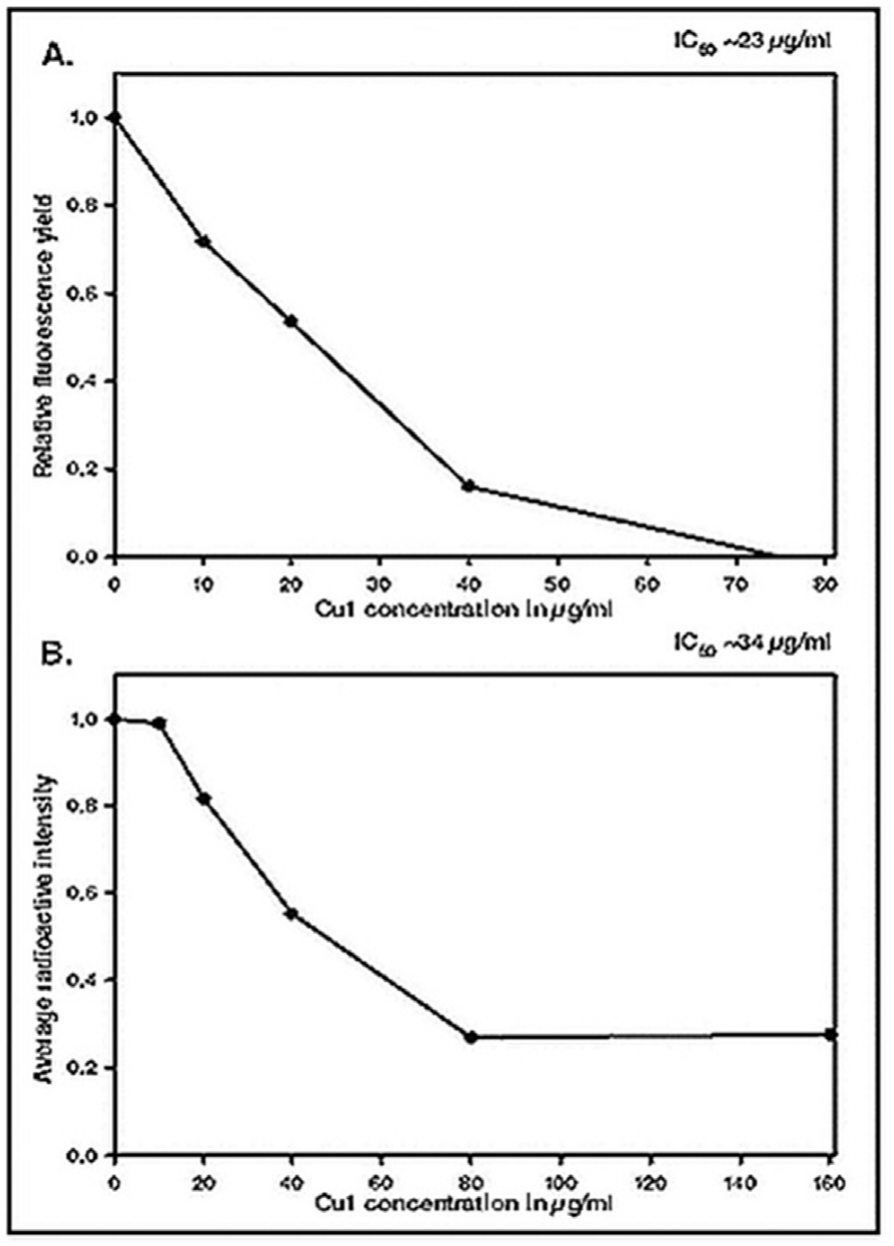
Determination of IC50 of CU1 on transcription by *Mtb* RNAP: **A.** Fluorescence based in vitro transcription assay: A plot of fluorescence intensity of RiboGreen bound to RNA in the presence of different concentrations of CU1 (in μg/ml).The estimated IC_50_ value of CU1 was found to be ∼23 ​μg/ml. The intensities were observed at excitation and emission wavelengths of 500 and 525 ​nm, respectively. **B.** Radioactivity based in vitro transcription assay. Average band intensity of transcripts in each lane from the Radioactivity based in vitro transcription assay was plotted for each concentration of CU1.

In EMSA assay *Mtb* RNAP*σ*^*A*^holo was incubated with Cy5 labelled *sinP3* DNA to form RP_o_, in presence of increasing concentrations of the repressor CU1. The products were resolved in 5% native PAGE and visualized by fluorescence scanning. The results show that RPo formation reduced significantly with the increasing concentrations of CU1. The intensity of the shift denoting RP_o_ formation started to reduce in presence of 20 ​μg/ml of CU1 and eventually being completely absent in the presence of 80 ​μg/ml CU1 in the reaction (figure-9B). This result showed that the transcriptional process was inhibited by CU1 at the initial open complex formation step of transcription, thereby causing transcriptional repression.

**Fig. 9B:**
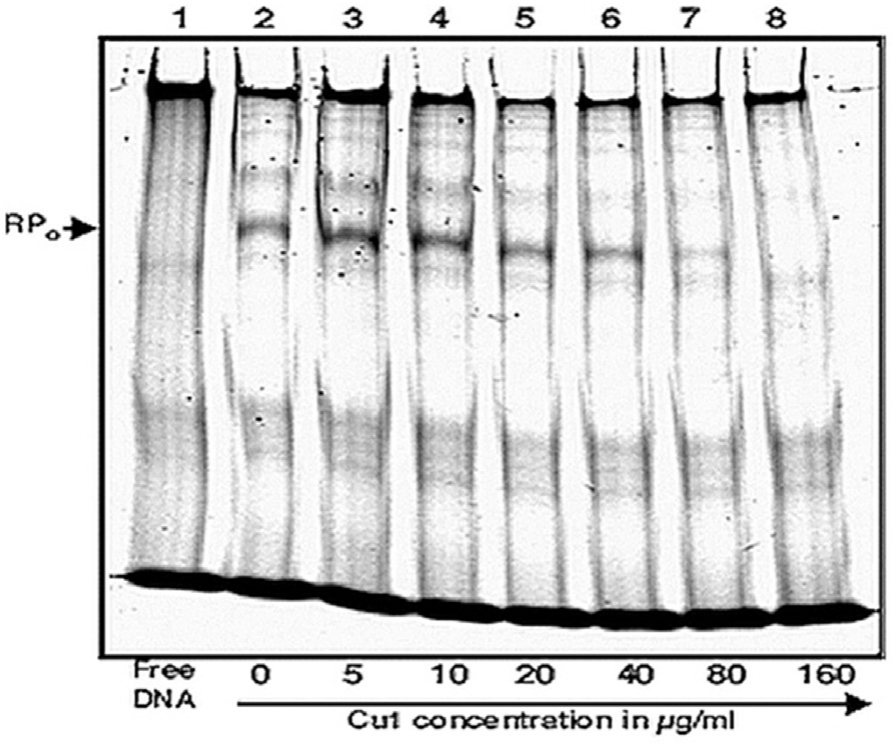
*Electrophoretic Mobility Shift Assay* (EMSA): EMSA of the RPo with Cy5 labelled *sinP3* promoter DNA fragment in the presence of increasing concentrations of CU1. For RP_o_ formation,1200nM Mtb RNAP *σ*^*A*^holo was incubated with 400 ​nM Cy5 labelled *sinP3* DNA and heparin (0.25 ​mg/ml) along with increasing concentrations of the repressor CU1 in subsequent lanes. Products were resolved by running in 5% native PAGE. Lane 1: Free DNA, Lanes 2–8: RP_o_ formation in presence of Cy5 labelled *sinP3* DNA and increasing concentrations of CU1 (in μg/ml), up to 160 ​μg/ml.

## 4 Discussion

We showed that ethanol extract of Cassia fistula bark was a nice ayurvedic drug against MDR bacteria. Specifically we demonstrated that CU1 target was RNA polymerase but yet to determine the target for CU3 phyto-chemical. However, CU1-drug had solubility problem as it had poor solubility in water. Ancient drug development programmes were based on phyto-extracts. But after the discovery of multiple antibiotics in 1930s and their commercialization in 1940s pushed back auyrvedic drugs development. Penicillins, tetracyclines, fluoroquinolones and aminoglycosides drugs saved mankind from cholera, typhoid, TB and other fungal and parasitic diseases between 1930 and 1980 (Chakraborty., 2019). Afterwards, we needed new derivatives of the drugs as penicillinases, oxacillinases, acetylases, phosphotransferases and adenyltransferases multidrug-resistant proteins appeared in plasmids as well as many drug efflux proteins like TetA/B/C, AcrAB, MexAB/CD/EF and MacAB (Chakraborty et al., 2017, 2018).

Multi-drug resistance has created a horror and phyto-drugs discovery has started again. Research advanced quinine, artiminisin, taxol, etoposide phyto-chemicals eliminating drug resistant malaria and cancer. This indicated a popular come back of phyto-drugs which are non-toxic and cheaper than synthetic antibiotics. We searched 80 plants to get new phyto-chemicals against multi-drug resistant bacteria. We pinpointed in previous papers that *Suregada multiflora* and *Cassia fistula* organic extracts were potential source of anti-bacterial phytochemicals (Chakraborty., 2015; Chakraborty et al., 2018). In this communication, We proved that one abundant *Cassia fistula* photo-chemical (CU1) has RNA Polymerase target and we substantially gave UV–Vis, MASS, NMR and FTIR evidences for structure prediction of the photo-chemical as terpentine linked to polybromophenols. MDR-TB is increasing in India and drug resistant mechanism in *Mycobacterium tuberculosis* is complex. We have showed that *Mycobacterium* RNA polymerase was the target of CU1 with IC50 ∼20–30 ​μg/ml. Interestingly, *Mycobacterium tuberculosis* plasmids were rare and TB-specific drugs were different supporting authentic RNA polymerase inhibition by CU1 phyto-chemical led to a new drug discovery against TB. We coined the name “MDR-CURE” which was a mixture of five medicinal plants extracts with traditional anti-oxidant and anti-inflammatory principals from 50% ethanolic solution of Neem bark and Haldi ryzome (Chakraborty., 2015, 2017a). When we injected MDR bacteria into rats, ampicillin or cefotaxime failed to clear infection but MDR-Cure cleared the 80–90% bacterial load in blood within two days.

Our method of CU1 isolation is cheap and any one can make 90% pure phyto-chemical for human use by simple TLC method. In truth, heterogeneous phyto-chemicals will be a new treatment method to conquer MDR pathogenesis and the herbal drugs are non-toxic. Due to multi-resistance and worldwide contamination of MDR bacteria in water and air, WHO predicted a GDP loss of 3% worldwide in the coming decade. In such scenario, Corona infections have created panic worldwide claiming 600 million infections and >2800000 deaths (Vaillancourt et al., 2020; Cantón et al., 2020). In India, peoples are dying in fever, jaundice, diabetes, asthma and cancer due to lack of treatment during corona virus pandemic and drug void. Such a pandemic is rare and if we use herbal medicine, many lives may be saved in poor and developing countries of Asia, Africa and Latin America.

MDR-infections as well as MDR-TB and MDR-Typhoid have soared recently in India as well as other countries due to antibiotics failure (Chakraborty., 2018; Chakraborty & Roy; 2020; Cole and Barnard., 2020). Thus, we claim heterogeneous phyto-antibiotics (CU1+ CU3+NU3+VU3 etc) may be resourceful and safer ayurvedic drug against MDR-bacteria. Multiple phyto-chemicals may also help to overcome the action of Tet, Acr, Mac and Mex types drug efflux proteins and twenty types of beta-lactamases, acetyltransferase and phosphotransferases enzymes (blaOXA, blaCTX-M, blaNDM1, AacC1/A1 and AphA2 inactivating penicillins (amoxicillin, cefotaxime), aminoglycosides (kanamycin, amikacin) and fluoroquinolones (ciprofloxacin) (Tiri et al., 2020; Chakraborty., 2018; Chakraborty et al., 2020).

## 5 Conclusion

Repeated oral antibiotics uses have been reported worldwide which drives more *mdr* gene creation and drug void. Thus, the need of MDR-Cure herbal drug against MDR pathogenesis is necessary and timing. We have characterized *Cassia fistula* (golden shower) CU1 phyto-chemical as complex structure of turpentine linked to bromopolyphenol but complete structure yet to determine. We are in process of characterizing other phyto-chemicals from *Suregada multiflora*, *Jatropha gossipiifolia* and *Shorea robusta.* Such bark is available in large amount from a medium sized tree which can grow in unfertile land and no water treatment was found necessary during summer. Recently, taxol, artiminisin, and quinine type of phyto-drugs have used as 95% pure similar to antibiotics. Thus, our approached to purify and characterize the CU1 phyto-chemical in detail to formulate as MDR-Cure drug is justified. Pure TLC purified CU1 was very active and such drug could be obtained at low cost. We have selected five plants and spices to make MDR-Cure and such drugs in combination will be effective against pan drug resistant bacteria. Abundant CU1 antibiotic is novel but less water soluble and will also help to understand the molecular mechanism of RNA synthesis in bacteria as there are very few drugs available today for RNA polymerase inhibition study and drug design against MDR bacteria. Our present goal to make a water soluble derivative of CU1 so that we can determine MIC carefully and such drug will be much better and more comparable to rifampicin. Moreover, we want to discover the target of CU3 and Cassia fistula bark crude ethanol extract is a good ayurvedic drug for MDR bacteria.

## Ethical issues

No human patient was used. Rat and Molly fish experiments with CU1 were discussed with Institutional Ethics Committee.

## CRediT authorship contribution statement

**Asit Kumar Chakraborty:** Funding acquisition, Conceptualization, Bioactive assays and Analysis. **Sourajit Saha:** RNA polymerase kinetics. **Kousik Poria:** CU1 purification. **Tanmoy Samanta:** Spectroscopy Technologies. **Sudhanshu Gautam:** Testing 11 phytochemicals on E. coli RNA polymerase. **Jayanta Mukhopadhyay:** Funding acquisition, Writing – original draft.

## Declaration of competing interest

The authors declare that they have no known competing financial interests or personal relationships that could have appeared to influence the work reported in this paper.
